# Dog Behavior Co-Varies with Height, Bodyweight and Skull Shape

**DOI:** 10.1371/journal.pone.0080529

**Published:** 2013-12-16

**Authors:** Paul D. McGreevy, Dana Georgevsky, Johanna Carrasco, Michael Valenzuela, Deborah L. Duffy, James A. Serpell

**Affiliations:** 1 Faculty of Veterinary Science, University of Sydney, New South Wales, Australia; 2 Brain and Mind Research Institute, Faculty of Medicine, University of Sydney, New South Wales, Australia; 3 School of Veterinary Medicine, University of Pennsylvania, Philadelphia, Pennsylvania, United States of America; The University of Wollongong, Australia

## Abstract

Dogs offer unique opportunities to study correlations between morphology and behavior because skull shapes and body shape are so diverse among breeds. Several studies have shown relationships between canine cephalic index (CI: the ratio of skull width to skull length) and neural architecture. Data on the CI of adult, show-quality dogs (six males and six females) were sourced in Australia along with existing data on the breeds' height, bodyweight and related to data on 36 behavioral traits of companion dogs (n = 8,301) of various common breeds (n = 49) collected internationally using the Canine Behavioral Assessment and Research Questionnaire (C-BARQ). Stepwise backward elimination regressions revealed that, across the breeds, 33 behavioral traits all but one of which are undesirable in companion animals correlated with either height alone (n = 14), bodyweight alone (n = 5), CI alone (n = 3), bodyweight-and-skull shape combined (n = 2), height-and-skull shape combined (n = 3) or height-and-bodyweight combined (n = 6). For example, breed average height showed strongly significant inverse relationships (p<0.001) with mounting persons or objects, touch sensitivity, urination when left alone, dog-directed fear, separation-related problems, non-social fear, defecation when left alone, owner-directed aggression, begging for food, urine marking and attachment/attention-seeking, while bodyweight showed strongly significant inverse relationships (p<0.001) with excitability and being reported as hyperactive. Apart from trainability, all regression coefficients with height were negative indicating that, across the breeds, behavior becomes more problematic as height decreases. Allogrooming increased strongly (p<0.001) with CI and inversely with height. CI alone showed a strong significant positive relationship with self-grooming (p<0.001) but a negative relationship with chasing (p = 0.020). The current study demonstrates how aspects of CI (and therefore brain shape), bodyweight and height co-vary with behavior. The biological basis for, and significance of, these associations remain to be determined.

## Introduction

Domestic dogs exhibit an extraordinary degree of morphological diversity. Such breed-to-breed variability applies equally to the canine skull. Coppinger and Schneider [1] noted that the morphology of working dogs' heads clustered according to their breed's original purpose. This observation was later supported by a series of studies focused on cephalic index (CI: the ratio of skull width to skull length). CI is correlated with a tendency for retinal ganglion cells to be concentrated in a form of an *area centralis* rather than a visual streak [2]. This feature of short-skulled dogs means that they have more visual acuity in the centre of their visual field but less in the periphery. Dogs with a high CI are predicted to have optimal ability to detect movement in the periphery and are also more likely to follow a human pointing gesture [3], suggesting that the arrangement of retinal cells may link with aspects of canine social cognition. In addition, magnetic resonance images (MRIs) of brains across a range of dogs with different skull shapes revealed that the relative reduction in skull length compared to width is significantly correlated to a progressive pitching of the brain, as well as with a downwards shift in the position of the olfactory lobe [4]. Taken together, these lines of evidence suggest that CI may be associated with changes in the way dogs perceive stimuli and possibly process information. Since short-skulled dogs (the brachycephalic breeds, such as pugs and boxers) are the result of generations of highly selective breeding, the Roberts et al. study [4] suggests that the remarkable diversity in domesticated dogs' body shape and size appears also to have led to human-induced adaptations in the organisation of the canine brain. More recently, it has been reported that skull shape shows sexual dimorphism in some breeds of dog and that, as predicted by Coppinger and Schneider [1], CI clusters vary among breed groupings. These differences in skull shape and therefore brain shape may be associated with predictable changes in inherent behavior. There is also emerging evidence that body size is an important covariate of certain behaviors in dogs [5].

It is possible that a brachycephalic head shape (high CI) may be a by-product of human selection for neotenous behavioral characteristics or that dolichocephaly is a product of selection for hunting/chasing ability.

The current study therefore aimed to relate height, bodyweight and CI of breeds with the highest number of entries per year in the ANKC studbook to a comprehensive behavioral profile of each breed. For this purpose, we employed a unique database that surveyed 8,301 dog owners using the Canine Behavioral Assessment and Research Questionnaire (C-BARQ; http://www.cbarq.org) [6]. We then examined associations between such breed-specific behavioral profiles and independent estimates of breed CI based on skull measurements of 588 dogs.

## Methods

### Cephalic index (CI)

The method was designed to ensure that representative dogs of each breed were measured. With the owners' permission, we sampled six females and six males from each breed. Photographing the dogs was deemed non-invasive and the study was approved by the University of New South Wales Animal Ethics Committee. As expected, no distress was evident because the subjects were show dogs and so were well accustomed to being held by their owners and approached by unfamiliar humans, such as judges. Equal numbers of males and females were selected to overcome any sexual dimorphism. The choice of six as a minimum was arbitrary. To be included, dogs had to be two years of age or older and of show quality or from show-quality lines. Littermates of dogs that had already been measured were avoided to ensure that the attributes of a particular litter were not over-represented in the data. To be included, breeds had to:

1. be recognised by the Australian National Kennel Council (ANKC),
2. be owned by breeders registered with Dogs NSW
3. have had more than 30 puppies registered nationally with the ANKC in 2009.

**Figure 1:**
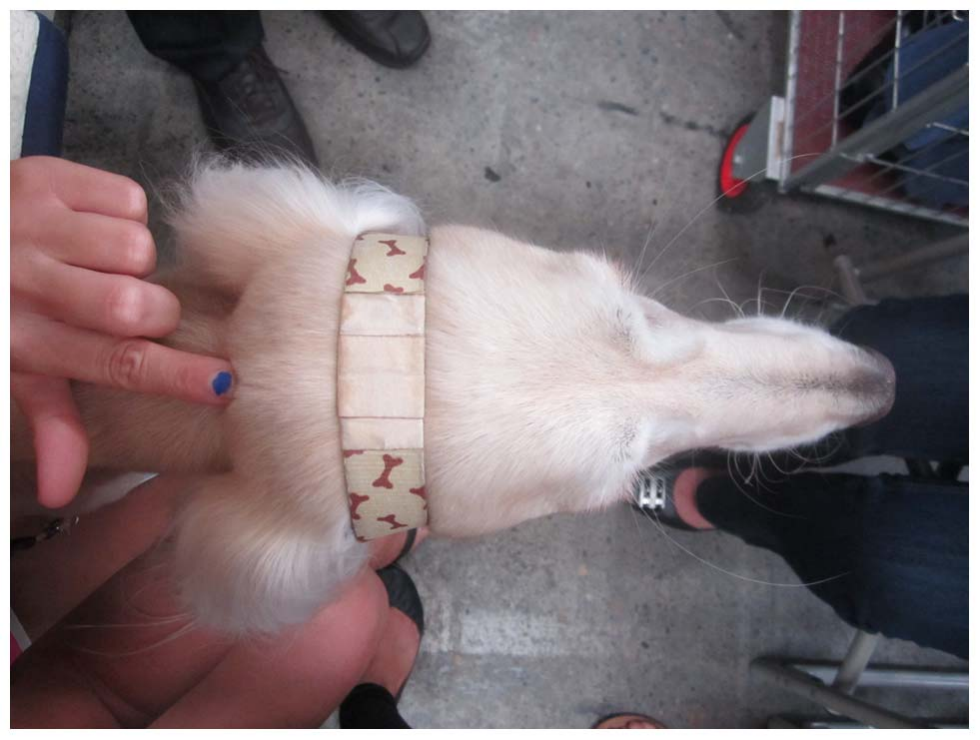
For the current study, each photograph was taken with the camera held horizontally, which allowed measurements to be obtained for each dog's skull length and width. The length was measured from the fingertip to the tip of the nose, and the width was measured from each zygomatic arch, which was displayed by the tape placed around the widest part of the dog's head.

We used an arbitrary threshold of 30 registrations per year to eliminate obscure breeds for which the Australian population may not be typical. Dogs were held by an assistant so that the nasal planum was horizontal and were then photographed using a dorso-ventral view of the top of the head, which allowed the length and width of the skull to be measured. A standardised cloth strap with a rectangular benchmark (2.5 cm×4.9 cm) was placed around the widest part of the dog's head. A finger placed on the occipital crest was placed and the photo was taken (*see* Figure 1) to permit post hoc measurement of the distance from the occipit to the most anterior point of the nose. The breed, dog's name and age were all recorded.

The team attended dog shows throughout New South Wales (NSW), Australia, from November, 2011, through to May, 2012. The majority of shows were held at the showgrounds at Erskine Park or Castle Hill, NSW, Australia. Breeds that had not been completely represented at shows over this period were then targeted at the Sydney Royal Easter Show. As this is the largest show in NSW, the breeds that were still not covered were excluded on the basis that their numbers were deemed too small to be representative of the breed as a whole. Using this process, we accumulated data on 80 breeds (n = 960 dogs).

Measurements were obtained using a GNU image manipulation program (http://www.gimp.org/) after normalisation to the reference rectangle. Cephalic index (CI) was calculated as 100× anterior-posterior skull width divided by skull length.

### Heights and weights of the breeds

The preferred heights for exhibition purposes were drawn from the ANKC breed standards, where available. Where different preferred heights were published for males and females in a given breed, the mean was calculated. For six breeds (Boston terrier, bulldog, chihuahua, miniature dachshund, Pomeranian and pug), preferred heights were not stated in the ANKC breed standards and so height data were instead drawn from a dog information portal: www.dogbreedinfo.com. Bodyweight data were drawn from the C-BARQ database (see below) of owners' reports on their dogs.

### C-BARQ

The C-BARQ is an owner-completed survey instrument designed to provide quantitative assessments of a wide array of behavioral characteristics of dogs, and has been widely used as a research tool for comparing behavior in different dog populations [6], [7], [8], [9], [10].The questionnaire consists of 100 items that ask respondents to indicate, using a series of 5-point ordinal rating scales, their dogs' typical responses to a variety of everyday situations during the recent past. Owners have been contributing data to the C-BARQ since 2002. The scales rate either the intensity (aggression, fear and excitability subscales) or frequency (all remaining subscales and miscellaneous items) of the behaviors, with a score of 0 indicating the absence of the behavior and a score of 4 indicating the most intense or frequent form of the behaviour. The C-BARQ currently comprises 14 behavioral factors or subscales, and a further 22 miscellaneous stand-alone items. Higher scores are generally less favourable for all items and subscales with the exception of ‘trainability’, for which higher scores are more desirable.

For the purposes of the current study, we drew data on 8,301 dogs of the breeds (n = 49) in the C-BARQ database that intersected with the breeds in our cephalic index study.

### Missing values

Dog owners may be unable to answer some of the C-BARQ questions for a variety of reasons. These non-responses are recorded as missing values and the subscale scores calculated as the average of the remaining completed item scores. If more than 25% of the items in a subscale are missing values, the subscale is not calculated.

The current version of the C-BARQ provides a set of quantitative scores for the following 14 different subscales or categories of behavior:

1. *Stranger-directed aggression*: Dog shows threatening or aggressive responses to strangers approaching or invading the dog's or the owner's personal space, territory, or home range.
2. *Owner-directed aggression*: Dog shows threatening or aggressive responses to the owner or other members of the household when challenged, manhandled, stared at, stepped over, or when approached while in possession of food or objects.
3. *Dog-directed aggression*: Dog shows threatening or aggressive responses when approached directly by unfamiliar dogs.
4. *Dog rivalry*: Dog shows aggressive or threatening responses to other familiar dogs in the same household.
5. *Stranger-directed fear*: Dog shows fearful or wary responses when approached directly by strangers.
6. *Non-social fear*: Dog shows fearful or wary responses to sudden or loud noises, traffic, and unfamiliar objects and situations.
7. *Dog-directed fear*: Dog shows fearful or wary responses when approached directly by unfamiliar dogs.
8. *Touch sensitivity*: Dog shows fearful or wary responses to potentially painful or uncomfortable procedures, including bathing, grooming, nail-clipping, and veterinary examinations.
9. *Separation-related behavior*: Dog vocalises and/or is destructive when separated from the owner, often accompanied or preceded by behavioral and autonomic signs of anxiety, including restlessness, loss of appetite, trembling, and excessive salivation.
10. *Attachment and attention-seeking*: Dog maintains close proximity to the owner or other members of the household, solicits affection or attention, and displays agitation when the owner gives attention to third parties.
11. *Trainability*: Dog shows a willingness to attend to the owner and obey simple commands. Dog is not easily distracted, tends to be a fast learner, responds positively to correction, and will fetch or retrieve objects.
12. *Chasing*: Dog chases cats, birds, and/or other small animals, given the opportunity.
13. *Excitability*: Dog displays strong reaction to potentially exciting or arousing events, such as going for walks or car trips, doorbells, arrival of visitors, and the owner arriving home; has difficulty calming down after such events.
14. *Energy level*: Dog is energetic, “always on the go”, and/or playful.

In addition, the C-BARQ provides frequency information on the occurrence of a further 22 miscellaneous problem behaviors, ranging from coprophagia to stereotypic spinning/tail-chasing. For further details about how subscales scores are calculated, see Duffy & Serpell [11].

### Linking Databases

For the current analyses, we used intersecting data from our CI measurements and the C-BARQ database. 49 breeds (see Table 1) had both CI data and 30 or more C-BARQ cases with which to generate a breed average profile across each of the 36 C-BARQ behavioral traits.

**Table 1:** The 49 breeds included in the current study and their preferred heights, mean CI and mean bodyweights as reported within the C-BARQ survey.

Breed (n for CBARQ data)	Preferred height for the breed (cm)	Mean reported bodyweight (kg ± S.D.; n)	Mean CI for the breed (± S.D.)
Akita (165)*	66.00	40.69±10.30 (181)	57.32±2.03
Alaskan Malamute (50)	61.00	37.14±10.12 (63)	58.08±2.30
American Staffordshire Terrier (65)	36.85	26.24±6.03 (73)	67.37±3.89
Australian Cattle Dog (225)	47.00	19.67±4.69 (255)	61.62±1.90
Australian Kelpie (45)	47.00	18.52±7.18 (52)	56.75±4.04
Australian Shepherd (379)	52.13	20.72±6.17 (419)	53.41±3.36
Basset Hound (48)	35.50	24.29±7.21 (59)	50.06±1.41
Beagle (165)	36.50	12.64±4.31 (205	59.77±2.76
Bernese Mountain Dog (164)	64.50	41.75±7.32 (178)	60.95±3.95
Bichon Frise (120)	30.00	6.90±2.55 (139)	63.55±4.76
Border Collie (418)	49.50	19.32±5.81 (473)	56.70±3.34
Borzoi (34)	71.00	31.69±7.45 (38)	38.66±1.67
Boston Terrier (53)	40.55	9.35±2.75 (69)	89.13±3.07
Boxer (194)	57.13	28.49±7.66 (224)	66.73±1.95
Bulldog (41)	35.50	23.26±5.77 (83)	86.61±3.73
Cairn Terrier (45)	29.50	7.92±2.09 (66)	63.43±3.59
Chihuahua (54)	19.00	3.37±1.63 (299)	71.90±3.14
Cocker Spaniel (American) (191)	39.25	12.73±2.80 (223)	59.42±6.40
Cocker Spaniel (English) (107)	39.25	12.73±2.80 (123)	48.88±2.52
Collie (Rough) (223)	56.00	28.13±6.57 (269)	46.64±3.24
Dachshund (Miniature) (74)	15.50	5.19±1.42 (87)	50.67±3.86
Dalmatian (84)	58.25	25.36±5.84 (104)	51.58±1.04
Doberman Pinscher (298)	67.00	33.19±7.23 (330)	46.28±3.05
English Setter (65)	65.00	25.19±5.56 (68)	43.57±2.42
English Springer Spaniel (129)	51.00	19.98±4.20 (147)	50.67±1.30
German Shepherd (704)	60.00	35.09±8.51 (822)	50.40±3.47
German Shorthaired Pointer (62)	62.25	26.38±6.49 (72)	49.50±3.26
Golden Retriever (554)	56.00	31.12±7.24 (652)	56.05±2.35
Great Dane (129)	73.50	57.59±11.66 (145)	56.59±4.77
Greyhound (114)	71.75	31.40±7.98 (120)	46.34±1.90
Irish Setter (60)	66.04	30.49±6.45 (58)	43.56±1.62
Italian Greyhound (40)	35.00	5.82±2.70 (41)	54.34±1.87
Jack Russell Terrier (220)	27.50	7.42±2.55 (253)	61.43±5.07
Labrador Retriever (1013)	56.00	32.11±8.37 (1185)	55.96±1.70
Maltese (97)	25.00	4.59±1.99 (114)	67.06±2.15
Miniature Schnauzer (108)	34.50	8.26±2.50 (132)	53.39±3.89
Papillon (52)	24.00	4.25±1.91 (64)	70.63±3.76
Pomeranian (111)	24.00	3.98±1.78 (148)	75.91±2.97
Pug (91)	30.25	8.98±2.86 (110)	98.54±4.52
Rhodesian Ridgeback (124)	62.00	38.22±6.33 (133)	50.43±2.06
Rottweiler (385)	63.00	42.32±10.03 (425)	63.58±6.29
Shetland Sheepdog (160)	36.25	11.14±4.99 (184)	50.46±2.40
Shih Tzu (160)	27.00	6.45±2.93 (153)	79.49±4.97
Siberian Husky (112)	55.00	23.95±5.73 (159)	54.88±3.31
Staffordshire Bull Terrier (142)	38.50	16.65±5.37 (188)	76.14±3.89
Vizsla (60)	59.00	23.64±6.52 (63)	49.82±2.47
Weimaraner (77)	62.75	31.98±6.45 (88)	49.95±2.97
West Highland White Terrier (61)	28.00	7.75±2.28 (75)	64.45±4.76

* Although there are two Akita breeds: The American and the Inu; C-BARQ offers only one choice: Akita.

### Statistical analysis

Stepwise backward elimination weighted regression analyses [12] were run manually for each of the 36 C-BARQ variables using GenStat Version 15 (VSN International, Hemel Hempstead, UK). For this regression, the data being analysed were C-BARQ variate means, and the weights used were the reciprocals of the squares of the standard error of the means, so N/SD^2^ (where N is the count of dogs and SD the standard deviation for each breed for which there were C-BARQ data).

First, a weighted regression was conducted with all three variables: CI, height and bodyweight were included. For Step 2, the least significant dependent variable, if any, was removed. For Step 3, the less significant of the two remaining variables, if any, was removed. The F-to-remove changed at each step depending on the degrees of freedom of the F test (but each being about 4.06). The final model containing any significant explanatory variables is reported in the summary table (see Table 2).

**Table 2:** Summary of significant (and marginally significant*) p-values and adjusted R^2^ values emerging from three stepwise backward elimination regressions that revealed relationships between CI, height, bodyweight and owner reports of 33 behavioral traits of companion dogs (n = 8,301) of various common breeds (n = 49).

	Cephalic Index	Height	Weight	R^2^
**Self grooming**	<0.001			23.8
**Chasing**	0.019^a^			9.2
**Dog-directed aggression**	0.057*			5.5
**Allo-grooming**	<0.001	<0.001		72.5
**Stranger-directed fear**	0.02^a^	<0.001		26.7
**Persistent barking**	0.01^a^	<0.001		35.1
**Compulsive staring**	0.032		<0.001	37.6
**Stealing food**	0.046^a^		0.002	16.4
**Mounting persons or objects**		<0.001		62.2
**Touch sensitivity**		<0.001		56.1
**Urination when left alone**		<0.001		51.5
**Dog-directed fear**		<0.001		46.4
**Separation-related problems**		<0.001		44.4
**Non-social fear**		<0.001		40.6
**Defecation when left alone**		<0.001		41.7
**Owner-directed aggression**		<0.001		39.1
**Begging for food**		<0.001		39.7
**Urine marking**		<0.001		35.3
**Attachment/attention-seeking**		<0.001		19.0
**Shadow/light chasing**		0.004		14.5
**Trainability**		0.005^b^		13.8
**Rolling in feces**		0.011		11.1
**Excitability**			<0.001	25.7
**Hyperactivity**			<0.001	21.1
**Dog rivalry**			0.003	15.1
**Escaping/roaming**			0.019	9.3
**Energy**			0.045	6.3
**Other stereotypic behavior**		<0.001	<0.001^c^	47.5
**Emotional urination**		<0.001	0.017^c^	27.0
**Tail chasing**		<0.001	0.004^c^	20.4
**Snapping at flies**		0.002	0.006^c^	15.0
**Stranger-directed aggression**		0.004	0.05^c^ *	15.0
**Nervous on stairs**		<0.001	0.027^c^	11.8

a Regression coefficients with CI were positive, apart from those with chasing, stranger-directed fear, persistent barking, and stealing food.

b All regression coefficients with height were negative, apart from the correlations of height with trainability.

c All regression coefficients with weight were negative, apart from the correlations of weight with other stereotypic behaviors, emotional urination, tail-chasing/spinning, snapping at flies, stranger-directed aggression and being nervous on stairs.

Empty cells denote the absence of any significant regression coefficients. The *adjusted* R^2^ value is a more reliable estimate of the amount of variation explained by an explanatory regression model.

Note that R^2^ in the summary table is the *adjusted* R^2^ value that is a more reliable estimate of the amount of variation explained by an explanatory regression model.

## Results

**Figure 2:**
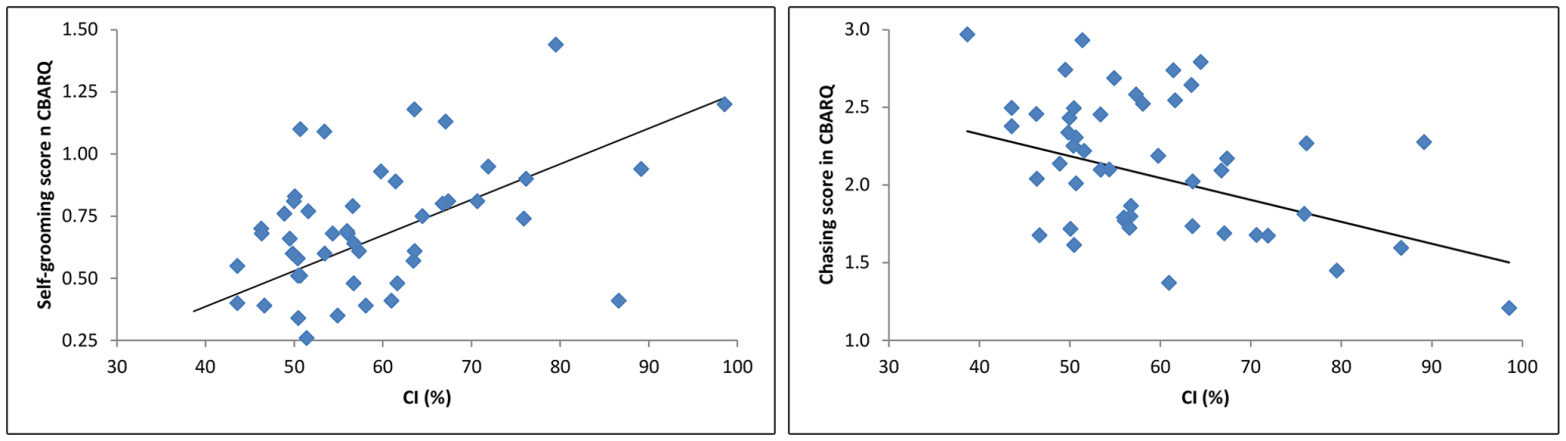
Plots of relationships between cephalic index (CI, expressed as a percentage) and self-grooming (a) and chasing (b). Trend lines represent the linear regression from the backward stepwise regression procedure.

Thirty-three behavioral traits had at least one significant predictor: height alone (n = 14), bodyweight alone (n = 5), CI alone n = 3), bodyweight-and-skull shape combined (n = 2), height-and-skull shape combined (n = 3) or height-and-bodyweight combined (n = 6). Regression coefficients with CI were positive, apart from those with chasing, stranger-directed fear and food stealing (see Figure 2). All regression coefficients with height were negative, apart from the regression coefficients of height with trainability. All regression coefficients with weight were negative, apart from the Regression coefficients of weight with other stereotypic behaviors, emotional urination, tail-chasing, compulsive snapping at flies, stranger-directed aggression, and being nervous on stairs. Three behavioral traits (coprophagia, chewing, and pulling on leash) showed no correlation with height, bodyweight or CI. As expected, a strong correlation emerged between preferred height (as dictated by the breed standards) and bodyweight for each breed (0.928; R-squared 86%).

## Discussion

The current study reveals previously unreported relationships between height, bodyweight, skull shape and behavior among dog breeds, and identifies particular canine morphotypes that are reliably associated with particular behavioral profiles. The results support the possibility that brachycephalic head shape (high CI) may be a by-product of human selection for neotenous behavioral characteristics or that dolichocephaly is a product of selection for hunting/chasing ability. They also suggest that data from the domestic dog may elucidate the biological processes responsible for behavioral and morphological diversification in other mammals. For example, paedomorphosis is considered an important mechanism forproducing evolutionary change [13], and domestic animals have been used as models of evolutionary change since Darwin [14].

It is worth noting that CBARQ data represent reports from the general population of owned dogs over six months of age. The ratio of reported males and females varies with breed. In this sense, the morphological and behavioural data are not perfectly aligned. The behavioral data from C-BARQ must be viewed with some caution because they are derived from owner reports. Owners may bring some bias to the process (e.g., they may focus on behaviors that they find particularly frustrating while ignoring others that are equally abnormal but less annoying). For example, persistent barking may be overlooked in a dog that repeatedly defecates when left alone. We also accept that some of the trends that emerge from C-BARQ may be the result of nurture rather than nature. For example, it is possible that some owners who acquire a dog purely as a companion may inadvertently reinforce attention-seeking behaviors because they find these, in the first instance at least, affirming [15]. Nevertheless, with such a large dataset and high levels of significance, the results are compelling and expose a series of fascinating links between behavior and morphology within a single species. Only three behavioral traits (coprophagia, chewing, and pulling on leash) failed to correlate with a morphological feature.

Some breed groups (such as terriers) are poorly represented in the current study. It would be appropriate to extend the coverage of breeds in the future. Using skulls rather than photographs of live dogs would both improve the accuracy of measurements and also make considerably more anatomical variables available for investigation. Catalogued collections of dog skulls such as those held by The Albert Heim Foundation for Canine Research in Switzerland could be used for this sort of study in future.

Several behaviors co-vary with one another. This has been reported elsewhere [7], [10] and may well reflect behaviors that arise from similar motivations. Similarly, some relationships with body size have been reported elsewhere [7], but neither to the extent nor in the detail revealed here. Generally, undesirable behaviors become more common or pronounced as height and weight decrease. However, an additional interaction with cephalic index is reported here. For allogrooming and compulsive staring, this risk is amplified in short-skulled dogs. For stranger-directed fear, barking persistently, and stealing food, it is amplified in long-skulled dogs.

The strong level of negative correlation between both height and bodyweight and a variety of problematic behaviors raises interesting questions and a variety of interpretations. For example, humans may be more tolerant of undesirable behaviour among small dogs and there may therefore be relaxed selection against such behavior and vice versa for bigger dogs, where this may be viewed as more potentially dangerous. Alternatively, higher rates of behavioral problems in small dogs may be environmentally induced by the ways in which people tend to keep them (e.g. over-indulged or over-protected). A further possibility is that selection for small body size is associated genetically with neurological changes in how dogs react to their environment, i.e. small dogs are innately more reactive and bigger dogs are more non-reactive. The current study shows that lighter dogs are especially likely to be reported as excitable, energetic and hyperactive. At least some of the behaviors more prevalent in shorter breeds (e.g. urination/defecation when left alone, separation problems, attachment/attention-seeking, and begging) could also be interpreted as infantile or care-soliciting behaviors, although whether these are the products of artificial selection for neotenous behavior (sensu [1]) or early environment [16] remains to be determined. All of these possibilities could, in theory, be tested.

Given that a strong correlation exists between preferred height (as predicated by the breed standards) and bodyweight for each breed, it is surprising that height and bodyweight correlate so discretely with behavioral tendencies such that attachment and fear are more features of short dogs, whereas energy, excitability and hyperactivity are features of light dogs. If smaller dogs are more likely to be kept indoors, these attributes may reflect the post-inhibitory rebound indoor dogs show when their owners return home or result from insufficient exercise emerging from an underestimation of the amount of off-lead exercise small dogs need [17]. Again, these possibilities could be tested.

The stranger-directed aggression seen in shorter dogs correlates inversely with bodyweight and could be an artefact of terriers clustering in the short-legged category. There may have been simultaneous selection for aggressive temperament (‘killer’ instinct) and short stature for chasing prey underground. Breeding history may act as a possible confounding factor with all or many of these associations [see 18, 19]. For example, if all bull-type breeds, or all terriers, or all miniature breeds share a common ancestor, the behavioral associations with brachycephaly, short legs or miniature size, respectively, may have been inherited from these ancestors. A cluster-based analysis of full genomes of these different breeds may prove helpful in this domain.

The behavioral responses associated with shortness alone include owner-directed aggression. It may, at first glance, be surprising to find that these dogs are high risk for attachment and attention seeking. Intuitively, one might not predict the same dogs aggressing against their owners and demonstrating attachment behaviors. However, the attention-seeking behaviors in this C-BARQ trait include pushiness and “jealousy” when attention is given to third parties. These are behaviors traditionally associated with resource guarding, so the association with owner-directed aggression may not be surprising.

It is worth nothing that among the short breeds are miniaturised versions of larger standard dogs (such as poodles) originally bred for purposes other than companionship. Small dogs may be retained as companions despite unwelcome behaviors more readily than larger dogs. So, the responses that correlate with CI rather than with body size may be especially noteworthy. Of these, only chasing showed a significant negative correlation with CI. At its simplest, this suggests that breeds selected for certain types of hunting or herding that involve visual pursuit of potential prey animals, tend to have long skulls, while those selected for companionship tend to have short skulls. Conversely, it implies that as humans selected dogs for short skulls and non-hunting traits, they sacrificed their tendency to hunt or simply found this less appropriate in companion dogs.

With eyesight that reflects the human tendency to have an *area centralis* rather than a visual streak [2], dogs with a high CI have greater acuity in the central visual field and may be more inclined than dolichocephalic dogs to attend to objects in front of them than those in the periphery. This may decrease scanning surveillance and help to account for their reduced chasing response.

Proposed as a model for human obsessive-compulsive disorder, tail-chasing in dogs typically has an early onset and variable manifestations [20]. It is associated with neutering, and is influenced by environmental factors such as deficiencies in maternal care and micronutrients [20]. Similarly, snapping at flies (which may or not be present) is commonly compulsive and possibly stereotypic [15]. It is interesting that these responses cluster with other responses that may indicate distress (notably stereotypic behavior and emotional urination) and correlate negatively with height but positively with weight. So, short stocky dogs are more at risk of showing so-called coping behaviors. The reason for this is unclear.

It is worth considering the behaviors associated with shortness in clusters because several of them may arise from similar motivations. This is part of the wider paradox of people wanting affirmative behaviors from their companion animals even though this may predispose the animals to separation anxiety and other signs of infantile dependence in the absence of their owners [21]. Begging and mounting behaviors are perhaps easier to tolerate in smaller-than-average dogs, but may both be reinforced by the owner's attention as well as by food and tactile rewards.

Smaller dogs may be permitted to show more undesirable responses than their larger counterparts simply because their behavior is likely to have less impact [7]. Developmental studies may help to reveal the role, if any, of early environment in the emergence of these unwelcome behavioral outcomes. It is clear from the current results that several of a breed's behavioral predispositions are strongly associated with its skull shape. The revelation that the size of the dog may have less influence on some behavioral outcomes than the shape of the skull is significant because it points to a possible relationship between neural architecture and behavior within a single species. It implies that CI and body size should be considered when judging whether a given dog's behavior is abnormal.

## Conclusions

The current study reveals previously unreported relationships between body size, skull shape and behavior among dog breeds, and indicates that particular canine morphotypes tend to be reliably associated with particular behavioral profiles. At this time it is unclear whether these associations between morphology and behavior represent functional co-adaptations or accidental by-products of allometric change, or even common branch points in their phylogenetic history. It is also impossible to determine from these data the extent to which the observed relationships are genetically or environmentally determined. Nevertheless, the current data remind us of the responsibility we have when selecting for extreme morphotypes, especially when these may change the behaviour of the animal. Hopefully, future taxonomic, genetic and developmental studies will help to clarify some of these issues. Overall, the findings suggest that the domestic dog represents a potentially valuable model for investigating the biological processes responsible for morphological and behavioral diversification.
